# Health-Related Quality of Life of Patients with Type 2 Diabetes Mellitus and Hypertension in Addis Ababa, Ethiopia

**DOI:** 10.4314/ejhs.v32i2.19

**Published:** 2022-03

**Authors:** Tariku Shimels, Rodas Asrat Kassu, Gelila Bogale, Mahteme Bekele Muleta, Gizachew Tadesse Akalu, Abrham Getachew, Zewdneh Shewamene, Melsew Getnet, Mebratu Abraha

**Affiliations:** 1 Research Directorate, Saint Paul's Hospital Millennium Medical College, Addis Ababa, Ethiopia; 2 Department of Neurology, Saint Paul's Hospital Millennium Medical College, Addis Ababa, Ethiopia; 3 United Vision Medical Services, Addis Ababa, Ethiopia; 4 Depertment of Microbiology, Immunology, and Parasitology, Saint Paul's Hospital Millennium Medical College, Addis Ababa, Ethiopia; 5 Ethiopian Health Insurance Agency, Addis Ababa, Ethiopia

**Keywords:** Addis Ababa, EQ-VAS, Ethiopia, EQ-5D-3L, HRQoL, Quality of life, T2DM and hypertension

## Abstract

**Background:**

The aim of this study was to assess the health-related quality of life of patients with T2DM and hypertension attending public health facilities in Addis Ababa, Ethiopia.

**Methods:**

A cross-sectional study was conducted from 1^st^ through 30^th^ August 2020 at the selected institutions. Health facilities were chosen purposively based on patient load. Participants were drawn after proportional to size allocation. A translated EQ-5D-3L, and EQ-VAS instrument was used to collect the data. Analysis was done using SPSS v.26.0. Both parametric and non-parametric models were applied in the analysis.

**Results:**

Of the 409 participants included, the majority were in the age group of 46–60 (36.0%), females (56.0%), from hospitals (54.8%), jobless (25.4%), and married (63.3%). Over two-thirds of the patients reported no problems with self-care, usual activity, and depression/anxiety. All dimensions showed an increasing proportion of moderate to severe problems in the age group beyond 45. Facility type, comorbid condition and age showed a statistically significant score difference for QoL. The overall prevalence of any problem was 59.0%. Education level, visit to a health center, and marriage showed lower odds of affected HRQoL whereas, lower monthly income and presence of comorbidities were opposite.

**Conclusion:**

HRQoL of patients in the study settings was suboptimal and below the general population. Attributes, such as education, facility type, marital status, income level, and comorbid status have a statistically significant association with HRQoL. Arrangement of a safe and quality health services is paramount, especially, during the COVID-19 pandemic.

## Introduction

According to the World Health Organization (WHO), quality of life (QoL) is defined as individuals' perceptions of their position in life in the context of the culture and value systems in which they live and about their goals, expectations, standards, and concerns (1). Another definition presents QoL as a global personal assessment of a single dimension that may be causally responsive to a variety of other distinct dimensions that encompasses the entire range of human experience, states, perceptions, and spheres of thought (2). When people's QoL is studied under specific health conditions, it is termed as health-related quality of life (HRQoL). Patrick and Erickson (1993) defined HRQoL as the value assigned to the duration of life as modified by the impairments, functional states, perceptions, and social opportunities that are influenced by disease, injury, treatment, or policy (3). The main aspect in a HRQoL study is how the manifestation of an illness or treatment is experienced by an individual (4).

Most HRQoL measurement approaches include two core concepts: subjectivity and multidimensionality. While the subjectivity emphasizes obtaining input from (or at least on behalf of) the affected person, the multidimensional aspect of the definition is a reminder that a full appreciation of the impact of illness and treatment requires an assessment of important life domains. It consists of at least three broad domains: physical, psychological, and social functioning, which are affected by one's disease and/or treatment (5). Accordingly, there are two main sets of instruments to measure HRQoL. One group is generic HRQoL measures which ask questions that are usually general enough to apply to almost everyone. Though more options are available in this set (3), the EuroQol five-dimensional (EQ-5D) standardized questionnaire (6) is being popular to score patient preferences, and generate index values. On the other hand, targeted instruments are applied to measure HRQoL for a specific disease such as cancer.

Studies reported the level of HRQoL in patients with type 2 diabetes mellitus (T2DM) at healthcare settings in Ethiopia (7, 8). A finding from Addis Ababa showed HRQoL to be influenced by diabetic nephropathic pain (9). However, adequate evidence on HRQoL or global quality of life (GQoL) for both T2DM and hypertensive patients, with or without concomitant occurrence, is scarce.

This study aims to present a comprehensive analysis of the HRQoL of patients with chronic non-communicable diseases (NCDs) in Ethiopia during the era of COVID-19. Potential factors associated with the affected HRQoL were also assessed.

## Methods

**Study setting, design and period**: This study was conducted in Addis Ababa, the capital city of Ethiopia. As per the 2007 national census (10), its total population was 3,384,569 with an annual growth rate of 3.8%. There are 12 public hospitals in the city (11), six of which and 103 health centers. This study was conducted at seven health facilities comprising of two hospitals namely; Saint Paul's Hospital Millennium Medical College (SPHMMC) and Ras Desta Damtew Memorial Hospital (RDDMH) as well as five health centers namely; Arada, Lideta, Nifas Silk Lafto Wereda 9, Akaki Kality and Bulbula health centers. A cross-sectional design was employed from 1^st^ throught 30^th^ of August 2020.

**Study population**: Adult outpatients with at least one chronic illness, have been taking medications for at least six months, visiting the facilities regularly, and those willing to participate in the study were included. Patients with any long term or temporal psychiatric problem, those admitted to the emergency/inpatient department, and patients aged below 14 were excluded.

**Sample size and smpling technique**: The single population proportion formula was employed to estimate the required sample size. The proportion of patients with affected HRQoL was assumed as 50%. A 95% confidence level and a 5% tolerable error were used. Considering a 10% contingency for nonresponse, the final required number of respondents was 423. We employed purposive sampling to include facilities whereas, participants were recruited consequitively. Allocation per T2DM and hypertensive cases was determined based on follow-up loads during the study period.

**Variables**: The dependent variables were HRQoL and GQoL, whereas the independent variables included sociodemographic (age, sex, marital status, education, occupation, substance use history, income level, type of relationship, number of people around), clinical(type of chronic illness, number of comorbidities, time since diagnosis and initiation of treatment), and type of follow-up facility.

**Instrument, procedure and quality management**: Data was collected using a structured interviewer-administered questionnaire. Patients' HRQoL was measured using the standardized EQ-5D-3L generic tool (6). The tool has five dimensions, namely: mobility, self-care, usual activities, pain/discomfort, and depression/anxiety on three levels; no problem, some or moderate problem, and severe problem. Patients' subjective judgment about their current state health was measured using the Euroqol visual analogue scale (EQ-VAS) (6) anchored with adapted patient aiding terms (12).

Data quality was ensured by training, continuous supervision, and the application of a pre-tested instrument. Furthermore, the instrument was translated to Amharic and back-translated to English for consitency. Content validity was checked by the study team. A Cronbach's alpha test was done to measure the reliability of the EQ-5D-3L scale on similar populations to health centres and hospitals (10% for each), and the score showed high internal consistency both for the hospitals (α=0.91) and health centres (α=0.85).

**Operational definitions**

**Affected HRQoL**: A cumulative score from mobility, self-care, usual activities, pain/discomfort, and depression/anxiety aspects of a patient and summing up to 6 or more on the EQ-5D-3L instrument. Score sums of 5 were considered not affected.

**Global quality of life (GQoL)**: Patients' self-rating of their own current health status on the EQ-VAS scale ranging from 0 (worst imaginable health state) to 100 (best imaginable health state).

**Any problem**: refers a patient's experience of at least one or a combination of problems to a moderate to severe degree on the EQ-5D-3L score.

**Ethical approval**: Ethical approval was obtained from Saint Paul's hospital millennium medical college (SPHMMC) and Addis Ababa regional health bureau (AARHB) research ethics committee. An informed verbal consent or ascent was sought from all study participants as appropriate. Data and information acquired through the study were kept confidential. No individual identifiers were collected and analysis was made in aggregate. Inclusion in the study was of voluntarily.

**Data analysis**: Data was coded, manually cleaned, and entered into SPSS version 26.0 for Windows. Both descriptive statistics binary logistics regression were used in data analysis. A 95% confidence level with a statistical significance of p≤0.2 (rule of thumb cut-off) for the bi-variable, and p≤0.05 for the multivariable model was used. A Shapiro-Wilk test was considered for checking the normality of GQoL scores. Because the data exihibited a non-parametric distribution (W(409)=0.94, p≤0.001), mean difference by sex, comorbid condition, and facility type were tested using the Mann Whitney U test. The potential difference by diagnosis type was tested using the Kruskal Wallis test. The ranked median difference test for Age category vs. GQoL was tested using Spearman's rank correlation coefficient.

## Results

**Sociodemographic profiles**: Of the 423 participants who fulfilled the inclusion criteria, 409 completed the investigator-administered questionnaire. The median age of respondents was 59 years (range: 19 to 95). About 229 (56.0%) were female and 224 (54.8%) were from hospitals. A total of 174 (42.5%) respondents were either able or not to read and write, whereas 165 (40.4%) attended primary or secondary education. About 104 (25.4%) were jobless and 97 (23.7%) were housewives. The majority were married (63.3%) and from the orthodox Christian faith (71.4%). The average monthly income ranged from 0 to 120000 with a mean (SD) of 2996.5 (6593) and a median of 2000 Ethiopian Birr (ETB). Over half (54.3%) of the patients are above the World Bank line of extreme poverty class (Table 1).

**Table 1:** socio-demographic characteristics of patients with T2DM and hypertension attending public health facilities in Addis Ababa, Ethiopia, August 2020 (n=409)

Characteristic	Category	Frequency	Percent
**Age** a			
	<=45 years	88	21.5
	46–60 years	147	35.9
	>=61 years	174	42.5
**Sex**			
	Male	180	44
	Female	229	56
**Facility level**			
	Health centers	185	45.2
	Hospitals	224	54.8
**Education**			
	Able/Not able to read and write	174	42.5
	Primary/secondary education	165	40.4
	College/University education	70	17.1
**Occupation**			
	Merchant	26	6.4
	Government employee	63	15.4
	Private employee	74	18.1
	Housewife	97	23.7
	Jobless	104	25.4
	Others b	45	11.0
**Marital status**			
	Unmarried	53	13.0
	Married	259	63.3
	Divorced/separated/widowed	97	23.7
**Religion**			
	Orthodox	292	71.4
	Protestant	44	10.8
	Muslim	61	14.9
	Others c	12	2.9
**Average monthly income (ETB)** d			
	<=1995	187	45.7
	>=1996	222	54.3
**Current history of any substance use** e			
	Yes	32	7.8
	No	377	92.2

a Is considered based on the average age for onset of t2dm in the sub-Saharan region (13)and Ethiopian age of retirement

b Includes students, daily laborers, and prostitutes

c Includes Catholic, Waqefeta, and Hawaryawi

d Is categorized based on the World Bank's poverty line definition for developing countries (14) and US Dollar exchange rate during the study period

e Refers to a patient reporting for having used any of either alcohol, Khat or cigarettes in the past three months.

**EQ-5D-3L measurement of patients' HRQoL**: The HRQoL of patients was measured using the EQ-5D-3L generic questionnaire. As shown in Table 2, majority of the patients do not have problems with mobility (261, 63.8%) self-care (323, 79.0%), performing usual activities (290, 70.9%), pain/discomfort (217, 53.1%) and depression/anxiety (280, 68.5%). Pain/discomfort (39.6%), mobility (31.3%), and depression/anxiety (28.6%) were the most frequently mentioned moderate problems. Only a few participants reported severe problems with each dimension.

**Table 2:** EQ-5D-3L frequencies reported by dimension and level among patients with T2DM and hypertension attending public health facilities in Addis Ababa, Ethiopia, August 2020 (n=409)

	Mobility	Self-care	Usual activities	Pain/ discomfort	Depression/anxiety
	N (%)	N (%)	N (%)	N (%)	N (%)
**Level 1**	261(63.8)	323(79.0)	290(70.9)	217(53.1)	280(68.5)
**Level 2**	128(31.3)	67(16.4)	91(22.2)	162(39.6)	117(28.6)
**Level 3**	20(4.9)	19(4.6)	28(6.8)	30(7.3)	12(2.9)

**Total**	409(100)	409(100)	409(100)	409(100)	409(100)

Key: Level 1: no problems, Level 2: some/moderate problems, Level 3: severe problems

The age-wise distribution of specific dimensions with three levels of the scale showed that the presence of any problems progressed with advancing age. Problems related to mobility, self-care, and usual activity were minimal compared to scores for pain/discomfort and depression/anxiety among the patients aged below 46. On the other hand, all dimensions showed an increasing proportion of moderate to severe problems in the subsequent age groups (Table 3).

**Table 3:** Age and sex-wise classification of EQ-5D-3L dimension and level among patients with T2DM and hypertension attending public health facilities in Addis Ababa, Ethiopia, August 2020

Variable			EQ-5D-3L scale				
	
			Mobility N (%)	Self-care N (%)	Usual activities N (%)	Pain/ discomfort N (%)	Depression/ anxiety N (%)
**Age**	<=45 (n=88)	Level 1	74(84.1)	86(97.70	81(92.0)	61(69.3)	66(75.0)
		Level 2	13(14.8)	1(1.1)	6(6.8)	23(26.1)	19(21.6)
		Level 3	1(1.1)	1(1.1)	1(1.1)	4(4.5)	3(3.4)

	46–60 (n=147)	Level 1	104(70.7)	123(83.7)	113(76.9)	91(61.9)	109(74.1)
		Level 2	39(26.5)	21(14.3)	26(17.7)	51(34.7)	36(24.5)
		Level 3	4(2.7)	3(2.0)	8(5.4)	5(3.4)	2(1.4)

	>60 (n=174)	Level 1	83(47.7)	114(65.5)	96(55.2)	65(37.4)	105(60.3)
		Level 2	76(43.7)	45(25.9)	59(33.9)	88(50.6)	62(35.6)
		Level 3	15(8.6)	15(8.6)	19(10.9)	21(12.1)	7(4.7)

**Sex**	Male (n=180)	Level 1	120 (66.7)	143(79.4)	128(71.1)	101(56.1)	132(73.3)
		Level 2	55 (30.5)	32(17.8)	43(23.8)	70(38.9)	44(24.5)
		Level 3	5(2.8)	5(2.8)	9(5.0)	9(5.0)	4(2.2)

	Female (n=229)	Level 1	141(61.5)	180(78.6)	162(70.7)	116(50.6)	148(64.6)
		Level 2	73(32.0)	35(15.3)	48(21.0)	92(40.2)	73(32.0)
		Level 3	15(6.5)	14(6.1)	19(8.3)	21(9.2)	8(3.4)

The pattern of HRQoL has also been assessed with respect to the gender of respondents. Overall, the proportion of males with no problems was higher for all dimensions compared to females, whereas, sever problems accounted for the reverse magnitude (Table 3).

**Figure 1:**
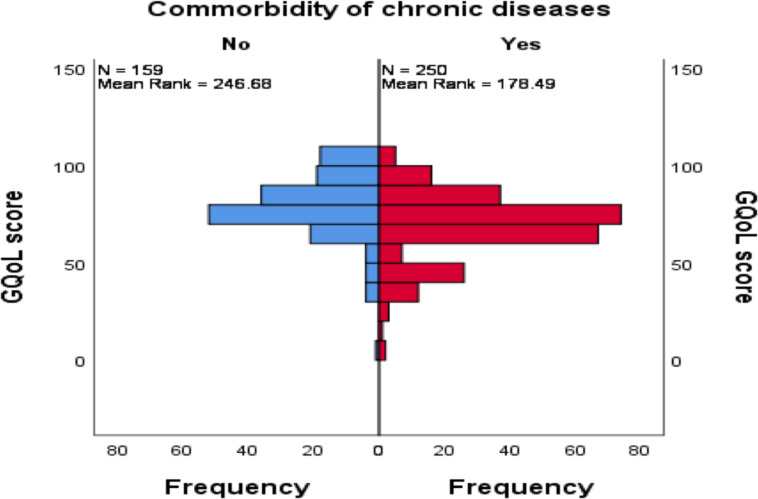
Mean rank difference of GQoL between single versus any additional chronic comorbidity among patients with T2DM and hypertension attending public health facilities in Addis Ababa, Ethiopia, August 2020

**Difference or correlation between GQoL score and patient attributes**: The mean (SD) score of GQoL was 69.44 (18.5) and the median was 70, with an interquartile range of 25 (85–60). The ranked mean difference for diagnosis type, sex, comorbidity, and facility type was tested against GQoL scores. No variation was noted by being under a specific diagnosis or combination, namely, T2DM only, hypertension only, or both (H (2) =4.282, P=0.118). A Mann Whitney test indicated that the distribution of GQoL is the same across both sexes (U=18722.000, P=0.106). Meanwhile, the same test has also shown a statistically significant rank difference between patients with single and any additional chronic comorbidities on GQoL score (U=13248.00, P≤0.001). Patients with a single chronic condition are more likely to have a higher mean rank of GQoL compared to those with any chronic comorbid conditions (Figure 1).

**Figure 2:**
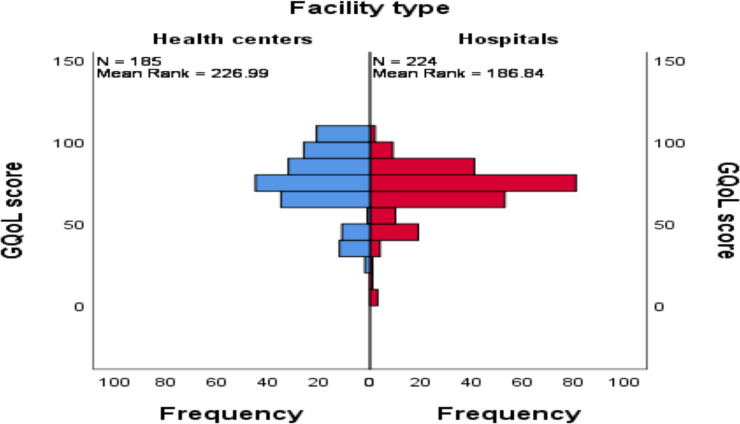
Mean rank difference of GQoL score between patients with chronic non-communicable diseases attended at heath centers versus hospitals in Addis Ababa, Ethiopia, August 2020

Similarly, the presence of mean rank difference was tested between health facility types over GQoL score distribution. A statistically significant difference was noted (U=16651, P≤0.001) where a higher rank of mean was scored among patients visiting health centers (Figure 2).

The correlation between age and GQoL of patients was tested using Spearman's rank correlation statistic. It was found that age showed a statistically significant negative correlation with GQoL score. The magnitude, however, is weak (*r_s_*=-0.27, p≤0.001).

**Affected HRQoL and associated factors**: Of all, 242 (59.2%) of the patients reported any problems based on the EQ-5D-3L questionnaire. This was regarded as affecting HRQoL, covering moderate to severe health-related problems on the scale. The remaining proportion of the patients has been reported to be in the best imaginable health state.

Compared to the age group 45 or below, the odds of developing any problems in the HRQoL dimensions was not statistically significant until the age of 60. About a 2.23 times higher likelihood of an affected HRQoL was noted in the subsequent age group (AOR: 2.23; 95% CI: 1.10–4.50). The odds of affected HRQoL among patients attending hospitals is higher by 75% (AOR: 1.75; 95%CI: 1.07–2.86) as compared to those visiting health centers. As education level increased, the likelihood of developing any problems decreased by 54% among those with primary education (AOR: 0.46; 95%CI: 0.27–0.81) and by 61% among those with college/university education (AOR: 0.39: 95%CI: 0.18–0.84) compared to those who are able to read and write. The odds of affected HRQoL among married patients were 60% lower as compared to the unmarried group (AOR: 0.40; 95%CI: 0.20–0.81). Being under the World Bank extreme poverty line (1.90USD per person per day) increased the odds of affected HRQoL by 77% (AOR: 1.77; 95%CI: 1.08–2.88). Similarly, presence of any chronic comorbidity increased the odds of affected HRQoL by 95% (AOR: 1.95; 95%CI: 1.22–3.14) (Table 4).

**Table 4:** Factors associated with affected HRQoL among patients with T2DM and hypertension attending public health facilities in Addis Ababa, Ethiopia, August 2020 (n=409)

Variable	Category	HRQoL		COR (95%CI)	AOR (95%CI)

		Affected (n)	Not affected (n)		
**Age**					
	<=45 years	40	48	1	1
	46–60 years	73	74	1.18(0.70–2.01)	1.03(0.55–1.90)
	>=61 years	129	45	3.44(2.01–5.90)	2.23(1.10–4.50)
**Facility level**					
	Health centers	99	86	1	1
	Hospitals	143	81	1.53(1.03–2.28)	1.75(1.07–2.86)
**Education**					
	Able/Not able to read and write	125	49	1	1
	Primary/secondary education	88	77	0.45(0.29–0.70)	0.46(0.27–0.81)
	College/University education	29	41	0.28(0.16–0.50)	0.39(0.18–0.84)
**Marital status**					
	Unmarried	32	21	1	1
	Married	134	125	0.70(0.39–1.28)	0.40(0.20–0.81)
	Divorced/separated/widowed	76	21	2.38(1.14–4.94)	0.98(0.42–2.30)
**Average monthly** **income (ETB)**					
	<=1995	130	57	2.24(1.49–9.37)	1.77(1.08–2.88)
	>=1996	112	110	1	1
**Comorbid** **condition**					
	No	71	88	1	1
	Yes	171	79	2.68(1.78–4.05)	1.95(1.22–3.14)

## Discussion

Evaluation of HRQoL of patients with chronic illness aids to understand the impact of an illness or the effect of an intervention (15). The present study showed that over half of the patients at the selected study units have an affected quality of life in terms of at least one dimension of the EQ-5D-3L instrument. About two-thirds of the patients reported no problems (Level I) with self-care, usual activity, and depression or anxiety. Pain/discomfort (39.6%), mobility (31.3%), and depression/anxiety (28.6%) were the most frequently mentioned moderate problems. This could be because such dimensions are frequently experienced among patients with chronic diseases (16). There is also a shred of evidence for mutual correlation and pathophysiologic mechanisms of pain and depression in this population (17). The study also identified that there is a notable influence that the COVID-19 pandemic posed on patients with chronic conditions in resource-limited settings (18). A high magnitude of COVID-19 related psychosocial distress was also reported by the general community (19). The distribution of moderate to severe levels of each dimension was noted to surge with increasing age. The fact that the majority was below the age of 60, after which functional and physical statuses are often reported to decline (20), may contribute to a higher average per dimension score. The fact that males have reported fewer problems and women experienced more with severe problems was in agreement with a report in the literature (21). A possible reason could be that women receive an extensive responsibility in households, and often suffer from socio-economic disparities in societies.

The mean GQoL score of patients in the present study (mean=69.44±18.5) is comparable with reports in a similar population (22). Slightly higher figures were documented from Japan (23) and India (24). Whereas comparators with the same measurement and population are lacking in the study area, the figure is smaller against the population-based EQ-VAS valuation score (mean=87.27±13.63) for Ethiopians (25). Yet, this result is almost the same as the EQ-VAS score of breast cancer patients (mean =69.94±20.36) at Tikur Anbessa Specialized Hospital in the same setting (26).

The mere co-presence of hypertension and T2DM may not be associated with an impaired quality of life (27). While co-occurrence is a common situation, this could be likely because it is often the further complications to come that affect a patient's HRQoL in both groups (28). However, there was a statistically significant difference between those with the single main diagnoses vs. co-presence of other comorbidities. The presence of reported complications such as stroke, heart disease, nerve problems, chronic kidney disease and other diseases such as chronic asthma might contribute to changes in QoL. Patients visiting health centers showed a better quality of life compared to those attending hospitals (mean rank=226.99 vs. 186.84). This could be attributed to differences in stage of the disease whereby patients with advanced states might be referred to hospitals. The fact that the majority of the patients from the hospitals reported at least one additional problem compared to their health center counterparts (68% vs. 53%) may support this argument.

On the other hand, attendance in hospitals may demand patients for long waiting times (due to high patient loads), less consultation time, and lower satisfaction with service, also documented elsewhere (29) and in Ethiopia (30). Likely, as hospitals were centers for COVID-19 management, patients might experience a greater degree of fear and anxiety, as the results showed (4.5% vs. 1.1%). Though skilled professional and technology capabilities are integral to being questionable in the present setting, a report also exists showing that better patient care services are delivered at community health centers (31). The age of patients exhibited a statistically significant negative correlation with the EQ-VAS score. Despite variation in tools used and diseases under consideration, studies support the inverse impact age has on patients' overall QoL (32).

In line with Spearman's correlation test result between GQoL and age, the multivariable logistic regression illustrated that lower age (below 60), in the present setting, showed a protective effect on the odds of affected health-related quality of life by 53%. This is likely as physical (33) and psychological (34) functioning diminishes with age, leading to comorbidities and the onset of complications (35). This also may highlight that routine psychological supports be integrated in NCD follow-up care. Set-ups with access to behavioral healthcare services improve patients' emotional dimension of health a study reported (36). Similarly, the affected HRQoL of patients decreased with higher education level and marriage. This accords with a report where educated and married (37) patients have reported having a better EQ 5D score, implying that they practice better prevention strategies and a healthy lifestyle. Conversely, the odds of affected HRQoL increased among patients visiting hospitals, those under the World Bank extreme poverty line, and the presence of any comorbid condition. Patients with extreme poverty conditions (those earning below 1.9 USD/per day per person) are less likely than others to engage in preventive and rehabilitative behavior. As most were either jobless, private employees or engaged in irregular income sources, the odds of affected HRQoL in this group can be understood as caused. While this holds among the general population (38) and other chronic diseases (39), patients with T2DM (40) and hypertension are affected most (41).

This study has tried to assess the HRQoL aspects of patients with T2DM and hypertension. Given these diseases often co-occur in most patients, the inclusion of varied socioeconomic diversity may also uncover the QoL of patients during the COVID-19 pandemic. However, results should be interpreted with caution due to lack of a probability sampling process and an unavailable validity report for the EQ-5D-3LVAS instrument among chronic patients in Ethiopia. Future studies with better design and probability sampling techniques are recommended.

Overall, about 59% of the patients have reported at least any problem based on the EQ-5D-3L instrument. Age showed a negative correlation with GQoL score. While education level, visiting a health center, and marriage were associated with lower odds of an affected HRQoL, the likelihood of the same outcome was increased with lower monthly income and presence of comorbidities. Enhanced pain management and psychosocial support systems could be integrated with NCD care in the study settings. It also appears that eligible and comfortable patients obtain follow-up services at the health centers. Understanding the emotional aspect of chronic illness, and updating all health professionals with the skills of psychosocial healthcare would be imperative. Further, a focused and holistic approach should be followed to improve QoL among those in the old age.
